# Fazekas score predicts cognitive decline & frailty in older adults: insights from the SAGE-AF cohort study

**DOI:** 10.1186/s42466-025-00439-3

**Published:** 2025-10-21

**Authors:** Bahadar S. Srichawla, Melanie K. Barbini, Darleen Lessard, Jane S. Saczynski, David D. McManus, Majaz Moonis

**Affiliations:** 1 Department of Neurology, University of Massachusetts Chan Medical School, Worcester, MA USA; 2 Department of Neurobiology, University of Massachusetts Chan Medical School, Worcester, MA USA; 3 Department of Medicine, University of Massachusetts Chan Medical School, Worcester, MA USA; 4 Department of Pharmacy and Health Systems Sciences, Northeastern University, Boston, MA USA

**Keywords:** Cognitive decline, Dementia, MoCA, Frailty, Small vessel disease, Fazekas score

## Abstract

**Background:**

Atrial fibrillation (AF) is a common condition in older adults, often associated with increased risks of cognitive decline and frailty. White matter hyperintensities (WMH), visible on neuroimaging and quantified by the Fazekas score, have been linked to both cognitive and physical impairments. However, the relationship between WMH, cognitive decline, and frailty in older adults with AF remains relatively underexplored.

**Methods:**

This study analyzed data from 86 participants in the SAGE-AF cohort, a two—year prospective multicenter cohort study of older adults with AF, who also had neuroimaging performed for clinical indications. WMH severity was assessed by independent reviewers using Fazekas scores from brain imaging. Cognitive function was measured using the Montreal Cognitive Assessment (MoCA), and frailty was assessed at baseline as well as 1- and 2-year follow-up visits by trained examiners as part of the SAGE-AF study protocol. Participants were characterized based on the severity of their white matter hyperintensities and compared to baseline and two-year cognitive and physical functional status. Longitudinal regression models were used to adjust for demographic, clinical, and geriatric covariates.

**Results:**

Participants with higher Fazekas scores (grades 2–3) demonstrated significantly lower baseline and follow-up MoCA scores and were more likely to meet frailty criteria over a two-year follow-up period. After adjusting for multiple factors known to influence cognitive decline, greater white matter hyperintensity (Fazekas grades 2–3) remained associated with a 2.6-fold increased risk of cognitive impairment at (*p* = 0.04) and a 2.7-fold increased risk of frailty at (*p* = 0.02).

**Conclusion:**

Higher Fazekas scores are related to cognitive decline and frailty in older adults with AF, emphasizing WMH as a critical biomarker for aging-related impairments. Neuroimaging tools like Fazekas scoring could enhance risk stratification and inform targeted interventions for this vulnerable population.

**Supplementary Information:**

The online version contains supplementary material available at 10.1186/s42466-025-00439-3.

## Background

As populations around the world age, the prevalence of cardiovascular diseases, cognitive decline and frailty have increased significantly, presenting major challenges for both public health and clinical care [1–6]. Cognitive decline encompasses a range of conditions, from mild subclinical cognitive impairment to more severe forms, such as dementia. Cognitive decline is frequently caused by aging-related neurovascular changes [7]. Frailty, a syndrome characterized by reduced physiological reserves and increased vulnerability to stressors, often coexists with cognitive decline and is strongly linked to the presence of comorbid cardiovascular diseases in older adults [8]. Understanding the mechanisms that link cognitive decline, frailty and heart health is crucial for improving clinical outcomes in aging populations.

Atrial fibrillation (AF) is a common cardiac arrhythmia in older adults and is associated with an elevated risk of stroke and cognitive decline due to cerebral hypoperfusion and thromboembolic events [9]. Individuals with AF are at an increased risk of developing both cognitive impairment and frailty. Given this association, exploring markers that reflect brain health in individuals with AF is particularly important for developing targeted interventions.

One potential marker that has gained attention for its role in both cognitive and physical decline is the presence of white matter hyperintensities (WMH) in the brain. WMH are commonly observed in magnetic resonance imaging (MRI) as hyperintense areas in white matter regions (e.g. centrum semiovale, corona radiata), often reflecting small vessel disease [10], and shown to be linked to stroke severity [11, 12]. The Fazekas score is a widely used tool to quantify the severity of WMH on brain MRI, ranging from mild punctate lesions to severe confluent lesions [13]. Higher Fazekas scores have been strongly associated with cognitive impairment, particularly in domains such as executive function, memory, and processing speed [14]. Moreover, emerging evidence suggests that WMH also contributes to the physical manifestations of frailty, given their impact on motor control and gait function [15]. Despite the established link between WMH and cognitive impairment, less is known about the impact of WMH on both cognitive decline and frailty in geriatric populations with atrial fibrillation (AF). Investigating the relationship between WMH, as quantified by the Fazekas score, cognitive decline, and frailty in individuals with AF is of particular interest.

This study aims to explore the association between the Fazekas score, cognitive impairment, and frailty in older adults with AF. In this prospective observational cohort study, we used data from the Systematic Assessment of Geriatric Elements in Atrial Fibrillation (or SAGE-AF) cohort. This study enrolled individuals with AF aged ≥ 65 years and followed them over 2 years. Using data from the prospective, multicenter SAGE-AF study, we seek to determine whether higher Fazekas scores are predictive of cognitive decline and frailty over a 2-year period. By elucidating the role of WMH in these aging-related conditions, this research may provide insights into potential interventions to mitigate the impact of cognitive and physical decline in vulnerable populations and potential mechanisms of cognitive and physical decline in AF. Because WMH and frailty are prevalent in community-dwelling elders irrespective of rhythm status, the present study confines itself to an AF cohort and does not address whether AF modifies these associations.

## Methods

### Design, setting, & participants

This study utilized data from the Systematic Assessment of Geriatric Elements in Atrial Fibrillation (SAGE-AF) study, a prospective, multicenter cohort study conducted from 2016 to 2018 across ambulatory care clinics in Massachusetts and Georgia, U.S.A. Eligible participants were aged 65 years and older, had a documented diagnosis of atrial fibrillation (AF), and a CHA_2_DS_2_-VASc score of ≥ 2. Exclusion criteria included contraindications for oral anticoagulant therapy, inability to provide informed consent in English, and an inability to participate in the 2-year follow-up period. Patients with significant thrombocytopenia, major bleeding, or those on anticoagulants for reasons other than AF were also excluded [16, 17].

Participants underwent comprehensive baseline assessments, including a medical history review, physical examination, and a 60-minute computer-assisted interview. Key demographic variables (age, sex, race, education), medical conditions (such as heart failure, diabetes, and hypertension), and behavioral factors (e.g., smoking status) were collected. Laboratory values, such as creatinine, hemoglobin, and platelet count, were also recorded. Stroke and bleeding risks were calculated using the CHA_2_DS_2_-VASc and HAS-BLED scores, respectively. The study was approved by the institution review board at each site. Of the 6507 subjects screened, 1244 participants were enrolled. From which only 86 subjects had an MRI or Computed tomography (CT) completed prior to enrollment. Thus all 86 subjects in this analysis had both neuroimaging data, and MoCA scores evaluated at enrollment, one-, and two-year endpoint. MRIs or CTs scans were completed if patients had concerns for neurological concerns, such as cognitive impairment.

### Medical record abstraction

Medical records were abstracted by trained research staff to ensure the accuracy and consistency of data collection. The abstraction process included reviewing and extracting relevant clinical information such as participants’ medical history, medication use, and laboratory values. Data collected included a history of intracranial hemorrhage, gastrointestinal bleeding, myocardial infarction, heart failure, hypertension, diabetes, renal disease, and other comorbidities. Medication use, particularly anticoagulants, antiplatelet agents, and antiarrhythmic drugs, was also abstracted from the records. Persistent AF was defined as continuous AF sustained > 7 days. Other data abstracted included relevant laboratory values (e.g., hemoglobin, INR, serum creatinine). CHA_2_DS_2_-VASc and HAS-BLED scores were calculated.

### Fazekas score and white matter hyperintensity assessment

WMH were assessed using MRI and CT scans of the brain obtained up to two years prior to participant enrollment in the SAGE-AF study. MRI images were obtained from a 1.5T MRI scanner (Signa Pioneer, General Electric Healthcare, Wisconsin, USA) using a standardized imaging protocol. WMH were evaluated on T_2_ weighted imaging. Fast gradient echo sequence as follows: TR/TE/flip angle = 7.5ms/3.0ms/8o, frequency/phase = 220/220, slice thickness = 1.2, reconstructed voxel size = 1.20 × 0.94 × 0.94 mm; sagittal 3D T_2_-FLAIR (Fluid Attenuated Inversion Recovery): TR/TE/flip angle/echo train length = 5400/maximum ~ 133/90o/140, frequency/phase = 256/224, slice thickness = 0.8 at 50% resolution, reconstructed voxel size = 0.80 × 0.49 × 0.49 mm. WMH severity was graded using the Fazekas scale by a board-certified neurologist (M.M.). The Fazekas scale is a widely used grading system to quantify WMH on a scale from 0 to 3, where:

- **Grade 0**: No white matter hyperintensities (normal white matter).
- **Grade 1**: Mild, punctate hyperintensities.
- **Grade 2**: Moderate, beginning confluence of lesions.
- **Grade 3**: Severe, large confluent areas of hyperintensity [13].

CT images were obtained using a CT scanner (Phillips, Spectral CT 7500) with a standardized imaging protocol. WMH were assessed using a previously published method [18] and imaged on axial 0.5 mm slices on the maximum intensity projection. A trained board-certified neurologist (M.M.) assessed WMH. The MRI Fazekas scale was adopted to quantify WMH on a scale from 0 to 3, where:

- Grade 0: No white matter.
- Grade 1: Periventricular cap lining or punctate foci.
- Grade 2: Periventricular halo or the beginning confluence of foci.
- Grade 3: Irregular periventricular lesions extending into the deep white matter with large confluent areas.

All baseline scans were additionally screened by a board-certified neurologist for other cerebral small-vessel-disease markers including prior cerebral infarctions and Boston 2.0 magnetic-resonance criteria for possible or probable cerebral amyloid angiopathy.

### Cognitive function assessment

Cognitive function, the primary outcome of interest, was evaluated using the Montreal Cognitive Assessment (MoCA), a commonly used screening tool designed to detect mild cognitive impairment [19]. The MoCA assesses various cognitive domains, including memory, visuospatial abilities, executive function, attention, concentration, working memory, language, and orientation. It consists of 30 items and takes approximately 10 min to administer. Scores range from 0 to 30, with lower scores indicating poorer cognitive function. A MoCA score of ≤ 23 was used as the cutoff for cognitive impairment, in accordance with previous studies on older adults [20]. The MoCA was administered at baseline, and again at 1-year and 2-year follow-ups to track cognitive changes over time.

### Frailty assessment

Frailty was assessed using the Fried Frailty Phenotype (FP), a validated instrument for measuring frailty in older adults [21]. This phenotype comprises five components:

- **Unintentional weight loss**: ≥5% of body weight lost over the previous year.
- **Exhaustion**: Measured through self-reported fatigue.
- **Low physical activity**: Assessed using standardized activity scales.
- **Slowness**: Evaluated by measuring walking speed over a set distance.
- **Weakness**: Determined using grip strength tests.

Participants meeting three or more criteria were classified as frail, those meeting one or two criteria were considered prefrail, and those with none of the criteria were classified as robust. Similar to the cognitive assessment, frailty was assessed at enrollment, one-year, and at two-years from enrollment.

### Statistical analysis

Descriptive statistics were used to summarize baseline demographic and clinical characteristics. Continuous variables were reported as means with standard deviations, while categorical variables were presented as frequencies and percentages. ANOVA and Chi-square tests were used for continuous and categorical variables respectively. Longitudinal binary mixed models were used to examine the association between Fazekas score and outcomes such as cognitive impairment and frailty, adjusting for potential confounders. Variables controlled for in the multivariate model included sex, age, race, marital status, AF duration, anticoagulation at baseline, CHA_2_DS_2_-VASc, HAS-BLED, heart failure, cardiovascular disease, peripheral vascular disease, hypertension, diabetes, stroke, current smoker, alcohol abuse, major bleed, cancer, liver disease, kidney disease, intercranial hemorrhage, cardiac surgery, valve repair, fall in the past 6 month, and depression. These variables were selected based on clinical relevance and significance (Table 1). Participants were grouped by Fazekas grade 0–1, and grade 2–3. All statistical analyses were conducted using SAS software, *version 9.4* (SAS Institute, Cary, North Carolina, USA). A two-sided *p*-value of < 0.05 was considered statistically significant for all analyses. In keeping with the a-priori aim of determining whether baseline white-matter hyperintensity burden predicts subsequent outcomes, we treated Fazekas grades 0–1 versus 2–3 as the primary exposure in longitudinal generalized linear mixed-effects models. More elaborate time-varying or cross-lagged frameworks were considered but judged under-powered for our cohort size (86 participants, three waves) and therefore were not pursued in the primary analysis.

**Table 1:** Baseline characteristics of participants stratified by Fazekas group (0–1 and 2–3). The table includes mean age, age categories (65–74 years and 75–84 years), and associated *p*-values indicating statistically significant differences between groups

Characteristics	Fazekas Group at Baseline		
Social Demographics	0–1 (*n* = 21)	2–3 (*n* = 65)	*p*-value
Age, mean, years (SD)	73.57	78.42	< 0.01
**Age**,** category (%)**			
65–74 years	66.67	33.85	< 0.05
75–84 years	23.81	40	
85 years and older	9.52	26.15	
**Female sex (%)**	57.14	50.77	0.61
**Race (%)**			
Non-Hispanic White	85.71	93.85	0.28
**Education (%)**			
College graduate or more	42.86	46.88	0.75
**AF Type**			
Paroxysmal	57.14	56.92	0.84
Persistent	23.81	18.46	
Permanent	4.76	10.77	
Other	14.29	13.85	
**Baseline (%)**			
Cognitive Impairment (MoCA ≤ 23)	23.81	46.15	0.07
**Frailty**			
Frail	9.52	12.31	0.02
Prefrail	38.10	66.15	
Not Frail	52.38	21.54	
Able to walk 15 ft	95.24	96.92	0.71
Depression	19.05	38.46	0.10
Anxiety	9.52	29.23	0.07
Social Isolation (MOS < 12)	0	10.77	0.12
**Smoking Status**			
Never smoker	57.14	41.54	0.27
Former smoker	38.1	56.92	
Current smoker	4.76	1.54	
**Bleeding history (%)**			
Intracranial hemorrhage	0	3.08	0.42
GI bleed	50	60	0.72
Major bleed	19.05	23.08	0.70
heart failure	28.57	44.62	0.19
CAD - MI or angina	0	33.85	< 0.01
Peripheral vascular disease	4.76	20	0.10
Hypertension	80.95	95.38	0.04
Type II Diabetes	33.33	21.54	0.27
Dyslipidemia	85.71	84.62	0.90
Ischemic Stroke	14.29	26.15	0.26
Alcohol Use	28.57	24.62	
Anemia	33.33	33.85	0.97
COPD	23.81	23.08	0.95
Renal Disease	38.1	41.54	0.78
Implantable cardiac device	9.52	33.85	0.03
Sleep apnea	28.57	36.92	0.49
**Blood levels**			
Creatine (mg/dL)	1.02	1.09	0.35
Hemoglobin (mg/dL)	13.44	13.29	0.49
Platelets	222.0	196.1	0.12
**Risk scores**			
CHA2DS2-VASc score (SD)	4.10	5.25	< 0.01
HAS-BLED score (SD)	3.29	3.74	0.08
**Medications (%)**			
Total #			
<=7	33.33	10.77	0.05
8–10	14.29	29.23	
11–13	28.57	21.54	
14+	23.81	38.46	
Any oral anticoagulants	90.48	81.54	0.33
Aspirin	14.29	40	0.03
Clopidogrel (Plavix)	0	10.77	0.12
Warfarin	63.16	71.7	0.49
Antiplatelet	0	10.77	0.12
**Treatment method**			
Rhythm control	47.62	52.38	0.91
Rate Control	66.67	78.46	0.27

### Ethical compliance with human study

This study was conducted in accordance with the principles outlined in the Declaration of Helsinki, which ensures the ethical treatment and protection of human subjects in biomedical research [22]. Prior to participation, all eligible participants provided written informed consent after receiving detailed information about the study procedures, potential risks, and benefits.

### Institutional review board approval

Ethical approval for the study was obtained from the Institutional Review Board (IRB) at the University of Massachusetts Chan Medical School. The IRB approval ensured that the study adhered to all regulatory and ethical standards for research involving human subjects. Additionally, appropriate measures were taken to protect participant confidentiality and ensure that all data were anonymized before analysis.

## Results

**Fig. 1:**
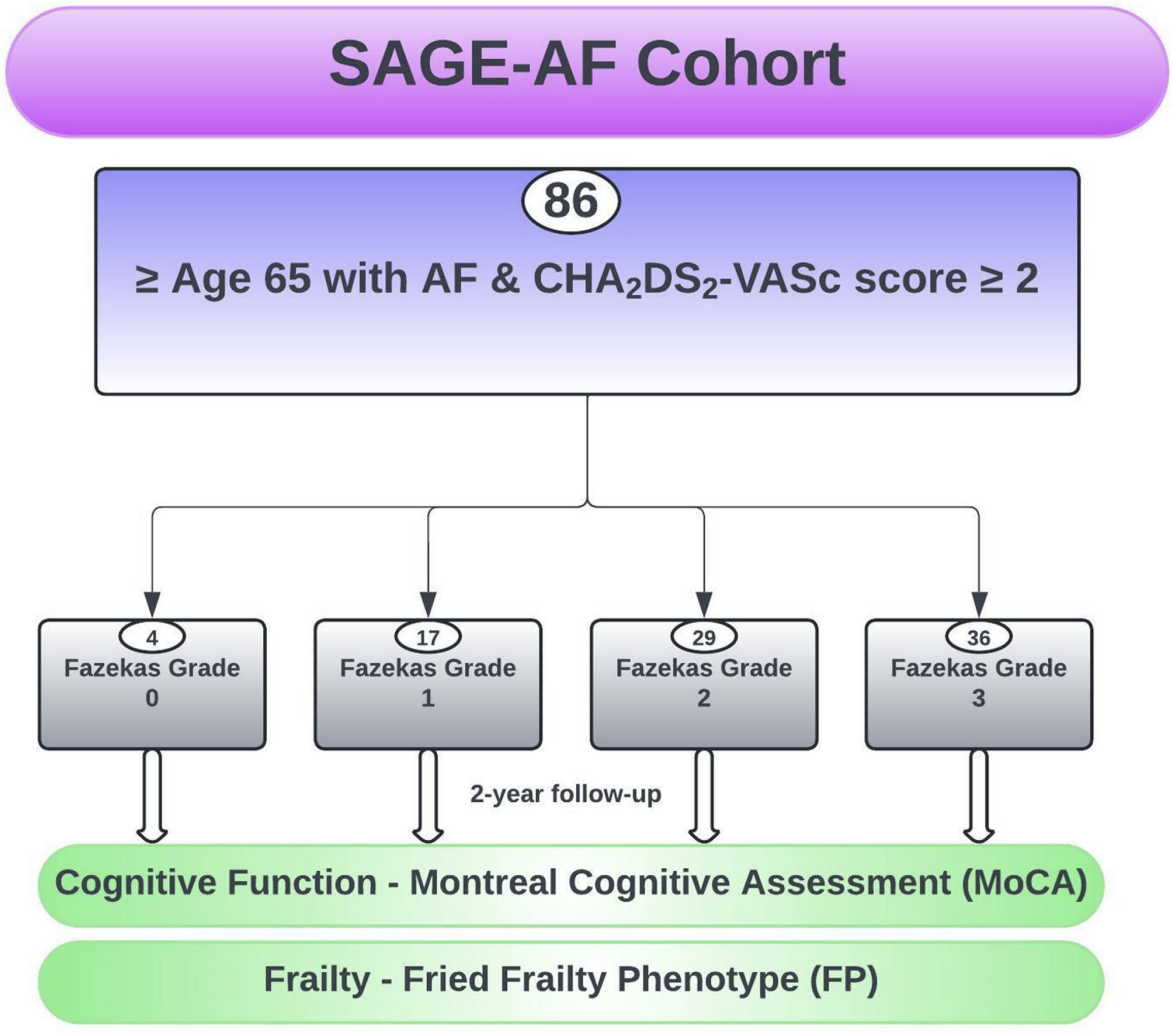
Flow diagram of included subjects from the SAGE-AF cohort with MRI completed prior to enrollment

A total of 86 SAGE-AF participants were included in this analysis. The total number of participants at baseline are included both at the 1- and 2-year follow-up period. A flow diagram of participant selection is provided in Fig. 1. Four participants had a Fazekas grade of zero, 17 Fazekas grade one, 29 Fazekas grade two, 36 Fazekas grade three. No silent (asymptomatic) cortical or sub-cortical infarcts were identified in any participant. Furthermore, none of the 86 participants fulfilled the Boston 2.0 magnetic-resonance criteria for possible or probable cerebral amyloid angiopathy. Baseline demographic and clinical characteristics are shown in Table 1.

The baseline characteristics of participants were analyzed by Fazekas group categories, with group 0–1 comprising 21 individuals and group 2–3 comprising 65 individuals. The mean age of participants in group 0–1 was lower (73.57 years) compared to those in group 2–3 (78.42 years), and this difference was statistically significant (*p* = 0.009). In terms of age distribution, a higher proportion of participants in the 65–74-year age category was found in group 0–1 (66.67%) compared to group 2–3 (33.85%), which was also significant (*p* = 0.027). Conversely, the proportion of participants aged 75–84 years was higher in group 2–3 (40%) compared to group 0–1 (23.81%), though no significant difference was observed for this category. These findings indicate that participants in the higher Fazekas group (2–3) were generally older and less represented in the younger age category.

**Fig. 2:**
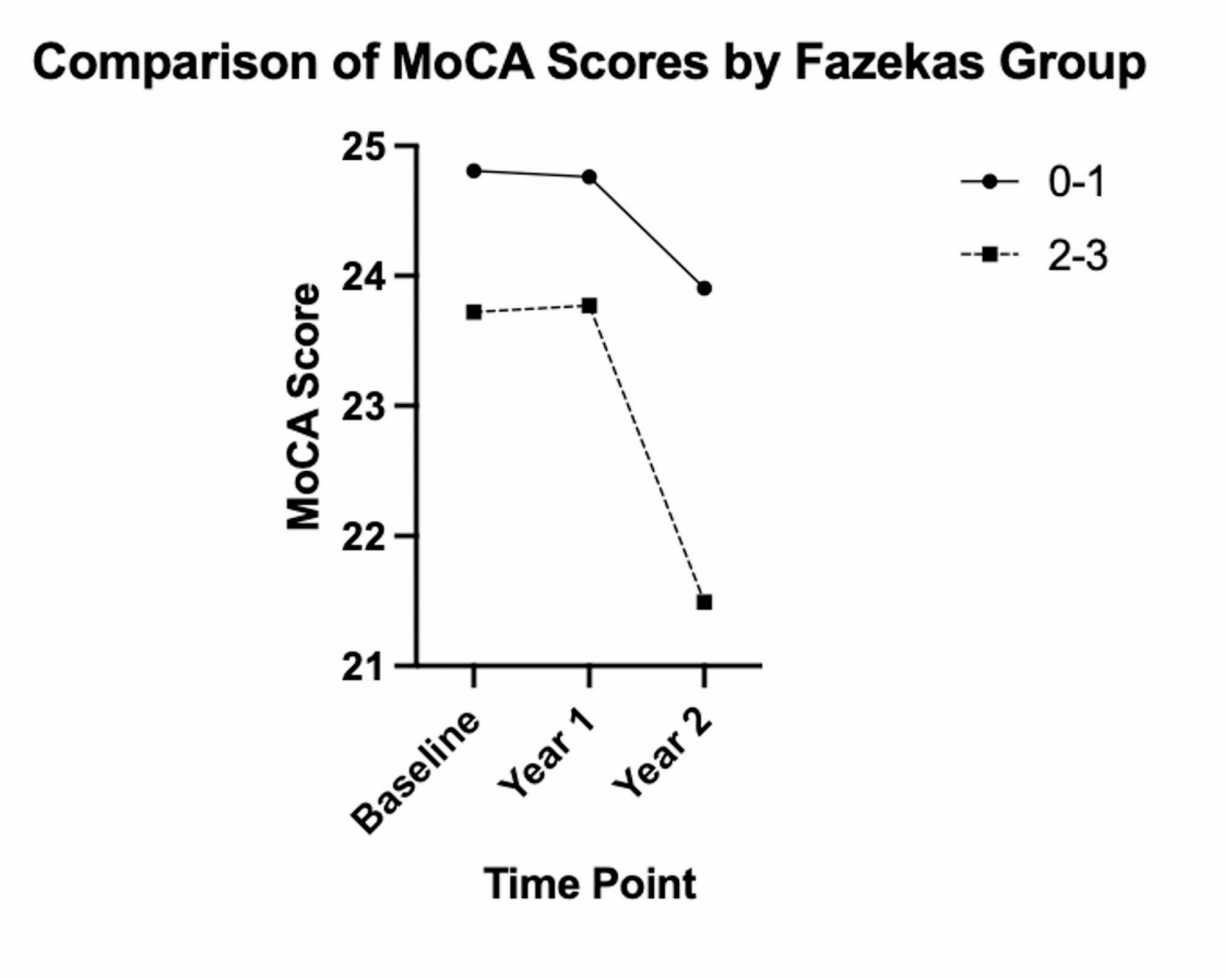
Longitudinal comparison of Montreal Cognitive Assessment (MoCA) scores by Fazekas group (0–1 and 2–3) over time. A nonsignificant drop in MoCA score was observed at the 2-year end point compared to baseline in the 0–1 Fazekas group (*p* = 0.76). A statistically significant drop in the MoCA score was observed at the 2-year end point compared to baseline in the 2–3 Fazekas group (*p* = 0.04)

**Fig. 3:**
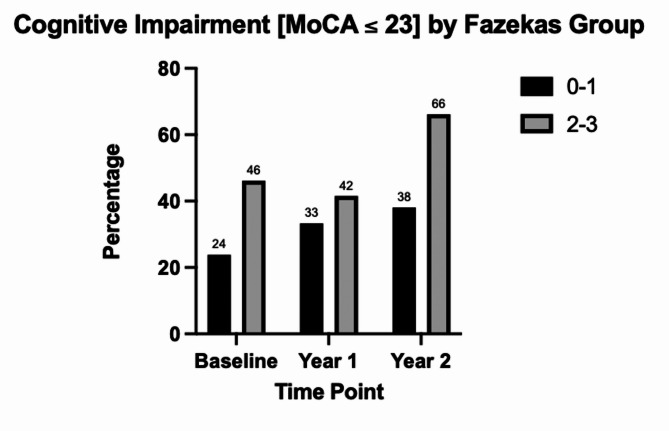
Percentage of individuals in the Fazekas groups (0–1 and 2–3) with cognitive impairment defined by a MoCA ≤ 23 at baseline, 1-, and 2-year end points. At baseline (*p* = 0.06) and at 1-year (*p* = 0.50) no statistically significant difference in cognitive impairment was observed between both groups. At year 2, a significantly larger proportion of individuals in the Fazekas group (2–3) are cognitively impaired compared to the Fazekas group (0–1) (*p* = 0.01)

The baseline MoCA score for Fazekas grades 0, 1, 2, 3 were 27.7, 24.2, 23.3, and 24.2 respectively. One-year follow-up MoCA scores for Fazekas grades 0, 1, 2, 3 were 26.5, 24.3, 23.4, and 24.1 respectively. Two-year follow-up MoCA scores for Fazekas grades 0, 1, 2, 3 were 26.0, 24.0, 21.6, 23.4 (Supplemental Figs. 1 & 2). After adjusting for clinical, demographic, and geriatric factors, individuals in the Fazekas grade 2–3 group were 2.62 times more likely to be cognitively impaired at the 2-year follow-up *(OR: 2.62 [95% CI] 1.02–6.7*,*p = 0.04).* After adjusting for clinical, demographic, and geriatric factors, individuals in the Fazekas grade 2–3 group were 2.68 times more likely to be frail at the 2-year follow-up *(OR: 2.68 [95% CI] 1.2–6.1 p = 0.02)*. **(**Figs. 2 and 3**)**.

## Discussion

In this observational study assessing cognitive function longitudinally, we found that higher Fazekas scores (2–3) were significant predictors of both cognitive impairment and frailty in older adults with AF over a two-year follow-up period, even after adjusting for clinical and demographic factors.

The Fazekas score is a radiological tool used to assess small vessel disease, an age-related pathological process that impacts cerebral microcirculation [18]. This condition manifests as symmetrical subcortical lesions or white matter changes, visible as hyperintensities on T_2_-weighted imaging or as hypoattenuation on conventional CT images [18]. Numerous studies have shown that the presence and severity of white matter lesions on MRI are predictive of cognitive decline and dementia in older adults [23, 24]. Moreover, white matter hyperintensities (WMHs) have also been associated with physical frailty in older adults.

AF, a heart arrhythmia affecting over 38 million people worldwide, causes inefficient cardiac blood flow, leading to blood stasis and clot formation, and significantly increases the risk of stroke [25]. Beyond stroke risk, AF is associated with a 20–30% increased risk of developing dementia [26, 27]. However, the exact mechanisms linking AF and dementia remain unclear [28]. Most studies have focused on AF-related strokes, cerebral hypoperfusion, and cerebral microbleeds as potential contributors to cognitive decline [29, 30,31, 32]. Silent cerebral infarcts, a form of brain ischemia without clinical symptoms, are common in AF patients and significantly increase the risk of future dementia [33]. Vascular pathology associated with AF may also contribute to the white matter lesions observed on imaging. Prior studies have identified macrostructural cortical abnormalities, microstructural anomalies of the cerebral white matter, and altered isotropic volume fractions in the cerebral cortex of AF patients [34]. Additionally, a Chinese study of acute ischemic stroke patients found that AF was associated with higher 12-month mortality and stroke recurrence, with outcomes worsening in the presence of imaging features such as lacunes, WMHs, and cerebral microbleeds [35]. Another study found that the presence of 10 or more cerebral microbleeds on MRI strongly correlated with subsequent intracranial hemorrhage, emphasizing the role of MRI in optimizing anticoagulation therapy for AF patients [36]. This study expands on prior research by integrating longitudinal data on atrial fibrillation, white matter hyperintensities, cognitive decline, and frailty, offering a comprehensive view of their interplay in older adults.

The utility of MRI and other imaging modalities in predicting cognitive decline and frailty is well-supported. For instance, combining MRI, PET, CSF data, and routine clinical tests significantly improves the accuracy of predicting Alzheimer’s disease onset compared to clinical testing alone [37]. Furthermore, increased WMH volume and more complex WMH shapes have been shown to correlate with frailty in older patients [38]. These findings suggest that MRI and CT imaging may serve as an imaging biomarker for early identification and intervention in aging patients, enabling the timely implementation of strategies to mitigate cognitive and physical decline. Imaging biomarkers could also facilitate treatment stratification and the development of targeted management strategies for AF patients based on individual MRI profiles.

Together, our results have several important clinical implications. First, incorporating routine screening of cognitive decline and frailty in the routine management of AF, particularly in older and high-risk patients, could be critical to assess and monitor. In addition, patients that may benefit from closer monitoring, geriatric assessment, or interdisciplinary care may be identified using risk stratification tools that utilize Fazekas score and other neuroimaging findings. For example, patients with higher Fazekas score may be more vulnerable to adverse outcomes such as falls, medication mismanagement, or challenges with anticoagulation adherence. These insights support a growing need for multidisciplinary collaboration between cardiologists, neurologist, and geriatricians to optimize AF care beyond rhythm and rate control. Future guidelines may consider incorporating cognitive and frailty assessments as part of a more holistic approach to managing AF in aging populations.

The strengths of this paper are rooted in its methodology and comprehensive data collection. The prospective collection of cognitive function reduced recall bias and enhanced the reliability of the findings by allowing for systematic and consistent evaluation of changes overtime. Moreover, the comprehensive frailty phenotyping provided a nuanced understanding of the physical and functional health of the cohort, allowing us to assess multiple dimensions of aging within our cohort. Another strength is the use of detailed brain imaging data with over-reads, allowing for added precision and accuracy in the evaluation of the neuroimaging markers. Lastly, the longitudinal study allowed us to observe temporal relationships and trajectories between cognitive decline, frailty, and WMHs.

However, several limitations should be considered. While the Fazekas score is traditionally determined using MRI, limited MRI availability in this cohort necessitated the use of CT imaging as a proxy. This approach is supported by prior literature demonstrating comparable Fazekas scores between CT and MRI [18]. Given that CT scans are more accessible, faster, and cost-effective, our study highlights their potential utility in evaluating Fazekas scores in AF patients. Nonetheless, the sample size limits the generalizability of our findings. A larger cohort would allow for stronger conclusions. Additionally, while MRI findings correlate with cognitive decline and frailty, causality cannot be established in this study. Although the MoCA test has been widely used as a screening tool for cognitive function it does not replace a battery of neuropsychological testing which would allow for a more robust analysis of cognition based on various domains, such as the Mini-Mental State Examination (MMSE), Clinical Dementia Rating Scale (CDR), Beck Depression Inventory (BDI-II), and comprehensive neuropsychological assessment results (CERAD-NAB battery). Therefore, we were unable to complete a phenotypic analysis of cognitive function. Future research should explore other covariates, such as AF type, duration, and treatment modalities, to refine predictive models. Our design lacked a non-AF comparator, precluding inference about the independent contribution of AF to the WMH–cognition–frailty pathway. Comparative studies including adults without AF are required to establish AF-specific risk amplification.

Future directions include conducting larger longitudinal studies to better understand the causal relationships between MRI findings and clinical progression. It would also be valuable to investigate whether interventions, such as rhythm control in AF or cognitive therapies, could mitigate MRI-related risk factors and improve outcomes in high-risk patients. Notably, AF patients on anticoagulant therapy have shown up to a 20% reduction in dementia risk [30, 31, 32]. Future investigations should incorporate voxel-wise lesion-symptom mapping or other region-specific techniques to isolate the cognitive impact of periventricular versus deep white-matter damage more precisely. Leveraging harmonized, high-resolution MRI in larger atrial-fibrillation cohorts will enable these spatially refined analyses and may uncover location-specific therapeutic targets. Combining MRI findings with other biomarkers could create comprehensive risk profiles for AF patients and guide personalized interventions aimed at reducing cognitive and physical decline. For example, analyzing demographic, clinical, neuropsychological and neuroimaging features has shown to help characterize patients with borderline cases of Alzheimer’s disease [39]. By leveraging imaging as a predictive tool, clinicians could better identify and treat at-risk patients, improving long-term outcomes. Although cortical atrophy is also known to contribute to cognitive decline, systematic quantification was not feasible here because volumetric T_1_-weighted MRI sequences were unavailable for a large fraction of participants (≈ 60% underwent non-contrast CT only). Consequently, WMH burden (Fazekas grade) remained the sole uniformly available neuro-imaging exposure in our primary models. Future prospective studies with harmonized MRI protocols should evaluate how cortical atrophy, WMH, and other small-vessel-disease markers jointly influence cognitive and functional trajectories in atrial fibrillation.

## Conclusion

This study highlights a significant association between higher Fazekas scores (grades 2–3) and increased risk of cognitive impairment and frailty over two years in older adults with atrial fibrillation (AF), independent of demographic, clinical, and geriatric factors. White matter hyperintensities (WMH), assessed using Fazekas scoring, emerge as a valuable biomarker for identifying at-risk individuals, with implications for integrating neuroimaging into routine care. While CT imaging served as a practical substitute for MRI in assessing WMH, larger studies are needed to validate these findings and explore causal mechanisms linking AF, WMH, and aging-related decline. Future research should investigate whether targeted interventions, such as cognitive therapies or optimized AF management, can mitigate these risks, enabling personalized care strategies to improve outcomes for aging populations.

## Supplementary Information

Below is the link to the electronic supplementary material.

**Supplementary Material 1**: **Supplemental Figure 1:** MoCA scores for Fazekas groups 0–3 at enrollment and one-year and two-year follow-up.

**Supplementary Material 2**: **Supplemental Figure 2:** Percentage of cohort with cognitive impairment (i.e. MoCA ≤ 23) for Fazekas groups 0–3 at baseline, one-year, and two-year follow-up.

## Data Availability

The datasets used and/or analyzed during the current study are available from the corresponding author on reasonable request.

## Abbreviations

AF: Atrial fibrillation.
CT: Computed tomography.
FP: Fried Frailty Phenotype.
MRI: Magnetic resonance imaging.
MoCA: Montreal Cognitive Assessment.
SAGE-AF: Systematic Assessment of Geriatric Elements in Atrial Fibrillation.
WMH: White matter hyperintensities.
