# Supplementary figures and images for: Fazekas score predicts cognitive decline & frailty in older adults: insights from the SAGE-AF cohort study
Source: Neurol Res Pract. 2025 Oct 21;7(1):78. doi: 10.1186/s42466-025-00439-3 (PMC12542089; doi:10.1186/s42466-025-00439-3)

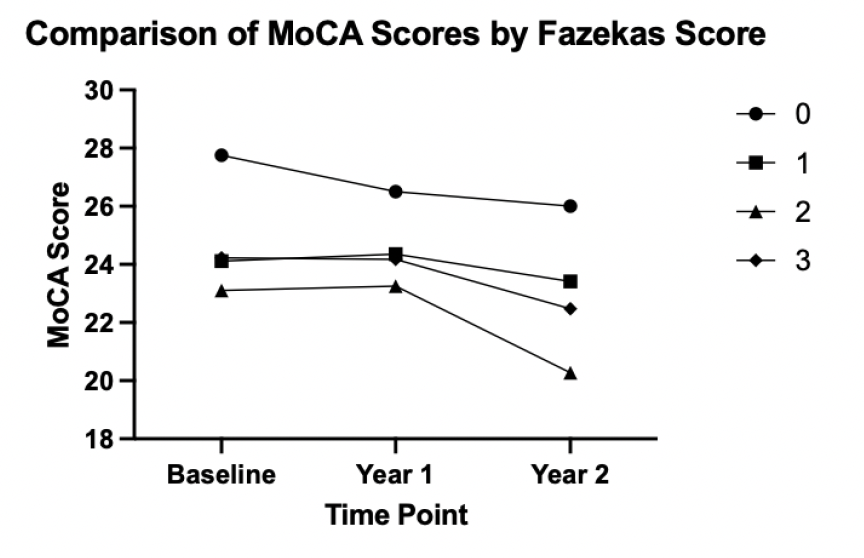

Supplement: Supplementary file 1 — Supplementary Material 1: Supplemental Figure 1: MoCA scores for Fazekas groups 0–3 at enrollment and one-year and two-year follow-up. [file 42466_2025_439_MOESM1_ESM.png]

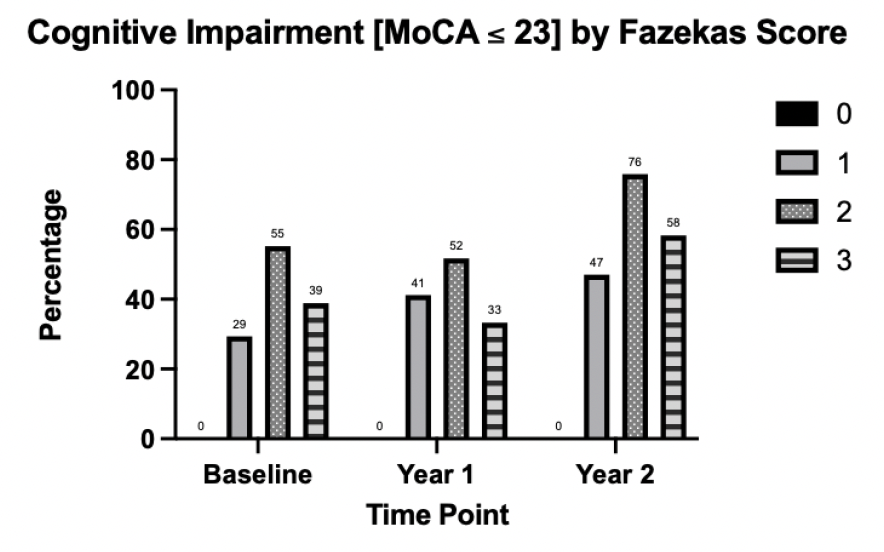

Supplement: Supplementary file 2 — Supplementary Material 2: Supplemental Figure 2: Percentage of cohort with cognitive impairment (i.e. MoCA ≤ 23) for Fazekas groups 0–3 at baseline, one-year, and two-year follow-up. [file 42466_2025_439_MOESM2_ESM.png]
